# Team-based Integrative Concept Map Student Presentations: An Active Learning Exercise

**DOI:** 10.1007/s40670-025-02495-5

**Published:** 2025-09-01

**Authors:** Aditi Kesari

**Affiliations:** Department of Medical Education, University of Tennessee Health Science Center, Memphis, TN USA

**Keywords:** Integration, Concept mapping, Active learning, Student presentations, Team-based activity, Peer teaching

## Abstract

Integration in medical education is critical for the students’ learning process. Concept mapping is effective at establishing linkages necessary for integration. This concept mapping exercise is developed to actively involve students in the integration process to enhance their understanding of topics while creating opportunities to foster other competencies such as teamwork, scholarship, peer teaching, and communication.

## Article

Integration in medical curricula allows for meaningful learning by breaking barriers between individual disciplines and emphasizing the relevance of topics to one another and their applications in clinical practice [1, 2]. Many of the MD programs in the US offer integrated curricula at their institutions and may implement one or more integration models, such as horizontal, vertical, or spiral integration. However, most of the integration effort seems to be driven by instructors and curriculum developers [1, 2]. This study provides a way to encourage student involvement in the integration process, primarily horizontal (integration between parallel disciplines) and vertical integration (linking basic sciences to clinical sciences), through an active learning exercise using concept mapping. Although concept mapping is an established tool that is often used in medical education to integrate concepts and convey integrative material [1, 3], this exercise is designed to achieve multiple other objectives. Besides promoting a self-directed approach in which students get to explore and identify the connections between topics themselves, this team-based exercise is also aimed at creating opportunities for fostering other skills and competencies such as teamwork, communication, peer teaching, and scholarship.

This pilot active learning exercise was designed for M1 students in the MD curriculum at UTHSC. Students were instructed to generate an integrative concept map in a team-based setting around a topic of their choice that was covered in the first 5 blocks of the Molecular Basis of Normal Body Function (MBNBF) course. The content covered in these blocks primarily included clinical applications of cell and molecular biology, genetics, biochemistry, and nutrition. Students were thus allowed to build a concept map around any unifying theme, such as an enzyme, a biochemical pathway, a clinical presentation or condition, etc., which could link the content from different sessions or blocks. Each concept map was required to have a title, a description/ rationale of that concept map (less than 100 words), the concept map itself, and references. Instructions were provided at least two weeks in advance, and each student team was required to submit their concept map by a set due date. This was followed by an in-class session in which the teams were expected to either volunteer or were randomly called out to present their concept maps to the entire class in 5–6 min. Formative feedback was provided at the end of the in-class session and in some instances after individual presentations. Finally, all the concept maps generated by students were made available to their classmates as additional study resources, with a disclaimer that these were not vetted by faculty.

Through this exercise, 30 integrative concept maps were generated in a team-based setting each year for the past three years and were made available to their respective classes as supplementary study resources. Thus, student teams were able to generate and disseminate their scholarly work to their peers in a low-stakes environment. Interacting with teammates and presenting concept maps to the entire class provided opportunities for peer teaching–learning experiences and a platform for communication. Visual and written communication skills were also fostered through the process of building and describing concept maps.

Moreover, the integrative concept maps generated by students brought forth connections between interdisciplinary topics covered in the MBNBF course, such as biochemistry and genetics (horizontal integration) and their clinical applications (vertical integration). In certain instances, linkages were also made with topics covered in other courses, such as the course covering anatomy and histology (horizontal integration).

Additionally, post-session survey data were collected for the past two years for continuous quality improvement purposes (Table 1). In 2023, 96 students recorded their attendance, and 74 responses were received on the post-session survey, whereas in 2024, 124 students recorded their attendance, and 101 responses were received. The survey data suggested that 96% of the total respondents were satisfied with the overall teaching–learning experience in 2023, whereas 93% of the total respondents were satisfied with the overall teaching–learning experience in 2024. Furthermore, 89% of the respondents in 2023 and 88% of the respondents in 2024 found the exercise of concept-map building in a team-based setting helpful for their learning. Additionally, 92% of the respondents in 2023 and 90% of the respondents in 2024 found the presentations of their peers to be useful in reinforcing the concepts covered in the course. The written comments from respondents further suggested that presentations by their peers helped them understand and reinforce concepts in a way that made sense to them and that they were able to see connections that they hadn’t noticed earlier.

**Table 1:** Post-session student survey data

Data collection year and number of responses	Response	Did you find the exercise of building concept maps with your teammates helpful?	Did you find presentations by your peers helpful in understanding/ reinforcing concepts covered in MBNBF?	Overall, were you satisfied with this teaching–learning experience?
**2023** **(74 responses)**	Yes	66 (89%)	68 (92%)	71 (96%)
	No	3 (4%)	2 (3%)	1 (1%)
	Not sure	5 (7%)	4 (5%)	2 (3%)
**2024** **(101 responses)**	Yes	89 (88%)	91 (90%)	94 (93%)
	No	6 (6%)	6 (6%)	3 (3%)
	Not sure	6 (6%)	4 (4%)	4 (4%)

Finally, here are a few points to consider for educators trying to adopt this activity in their curriculum. Some comments on the 2023 survey indicated that respondents would have appreciated it if this activity had counted towards their grades. In response to that, since then, teams submitting their concept maps in a timely fashion were given points, which counted towards their final grades. Secondly, while formative feedback was provided to students during the in-class session, the availability of additional resources (time/ faculty) to review all the concept maps and provide individualized formative feedback in a timely manner would have further enhanced the reliability of these resources. Another approach to this would have been to involve students in the peer review process. Finally, a couple of respondents mentioned that they would have preferred if the topics were assigned to them rather than having to choose on their own. One comment on this further elaborated that this was primarily due to their team dynamics. While we can acknowledge this feedback, the purpose of this self-directed approach to identify the topic of choice and build a meaningful concept map around it is to ensure that the students are involved in the integration process through and through. This approach enabled various connections to come forth, which other students had missed, as suggested in some comments mentioned above. Additionally, this feedback also emphasizes the need for opportunities where students can learn to navigate through team dynamics, and this exercise provided that opportunity.

In conclusion, this team-based concept map generation exercise empowered students to be involved in the integration process. The development and presentation of concept maps also created opportunities to foster peer teaching–learning experience, scholarship, teamwork, and communication skills in a low-stakes environment. Further studies are needed to evaluate the effectiveness of this exercise in promoting growth in these individual areas.

## Data Availability

The data supporting the findings of the study are included in the manuscript. Additional information may be made available by the author upon reasonable request.
